# Content Validation and Perceived Value of Text Messages to Promote Physical Activity Among U.S. Older Adults and Care Partners

**DOI:** 10.3390/ijerph23020258

**Published:** 2026-02-18

**Authors:** Oluwaseun Adeyemi, Tracy Chippendale, Gbenga Ogedegbe, Dowin Boatright, Joshua Chodosh

**Affiliations:** 1 Department of Emergency Medicine, New York University Grossman School of Medicine, New York, NY 10016, USA; 2 Department of Occupational Therapy, New York University Steinhardt School of Culture, Education, and Human Development, New York, NY 10003, USA; 3 Institute for Excellence in Health Equity, New York University Grossman School of Medicine, New York, NY 10016, USA; 4 Department of Population Health, New York University Grossman School of Medicine, New York, NY 10016, USA; 5 Department of Medicine, New York University Grossman School of Medicine, New York, NY 10016, USA; 6 Veterans Affairs New York Harbor Healthcare System, New York, NY 10010, USA

**Keywords:** motivational text messages, physical activity, item validation, older adults, care partners

## Abstract

**Highlights:**

**Public health relevance—How does this work relate to a public health issue?**

- Physical inactivity among older adults is linked to functional decline, falls, and chronic disease.
- This study addresses the need for scalable, low-cost strategies to promote physical activity in aging populations and their care partners.

**Public health significance—Why is this work of significance to public health?**

- By validating motivational text messages, this work establishes evidence-based tools that can be integrated into digital interventions to increase activity levels in older adults.
- The finding that messages are equally motivating for both older adults and care partners highlights the potential for dyadic approaches to improving health behaviors.

**Public health implications—What are the key implications or messages for practitioners, policy makers and/or researchers in public health?**

- Practitioners and health systems can incorporate these validated messages into remote monitoring platforms or fall-prevention programs to support routine physical activity.
- Policymakers and researchers can use this foundation to develop and test scalable, technology-enabled interventions that address physical inactivity among older adults.

**Abstract:**

Background: Motivational text messages can encourage increased physical activity. This study aimed to validate motivational text messages among older adults and care partners and to assess differences in perceived motivational value between the two groups. Methods: We designed nine motivational text messages to capture nine distinct physical activity scenarios. For this cross-sectional observational study, we enrolled 14 content experts, 310 older adults, and 305 care partners. Content experts assessed the relevance, while the older adults and care partners assessed the perceived motivational value of each text message on a 5-point Likert scale. We computed the item content validity index and assessed differences in perceived motivational value among older adults and care partners using quantile regression while adjusting for sociodemographic and health characteristics. Results: The item content validity index ranged from 0.86 to 1.00. The median (interquartile range) perceived motivational value for each text message was 4.0 (3.0–5.0), and there were no statistically significant differences in reported motivational values between older adults and care partners. Conclusion: We present nine content-validated text messages with high motivational value for older adults and care partners that can be integrated into technology-based intervention studies and may improve physical activity behavior in both groups.

## 1. Introduction

Physical inactivity among older adults is a major risk factor for frailty, functional decline, disability, and poor health outcomes [1,2,3]. As older adults age, reductions in strength, balance, and endurance can increase the risk of falls, loss of independence, and hospitalization and institutionalization [3,4,5]. Regular physical activity can mitigate these declines, improve mood, and enhance overall well-being. Interventions that combine aerobic, strength, and balance training produce the largest gains in independence and quality of life [6,7,8]. However, participation in recommended activity levels remains suboptimal among community-dwelling older adults, with many reporting only intermittent engagement due to barriers such as pain, fatigue, limited access to programs, and competing health priorities [9,10,11].

Technology-based interventions, including digital text messaging, offer scalable approaches to promote physical activity in aging populations [11,12]. These tools can provide real-time feedback in support of personal goals and social reinforcement, which are critical for sustaining motivation [13,14]. Among these technologies, automated text messages are a promising, low-cost strategy for delivering tailored encouragement and promoting self-management behaviors [15,16]. In the context of physical activity, motivational text messages can reinforce positive habits, promote self-efficacy, and foster accountability [17,18,19,20]. Despite increasing use [17,21], there is a paucity of evidence on how older adults and care partners interpret and respond to motivational text messages.

Using Self-Determination theory (SDT) [22], we formulated motivational text messages to promote physical activity among older adults and care partners. The SDT provides a well-established framework for understanding motivation and behavior change, particularly in the context of health promotion [22]. SDT posits that sustained engagement in health behaviors is most likely when three core psychological needs are supported: autonomy (a sense of choice and control), competence (feeling capable of success), and relatedness (feeling supported and connected) [23]. Among older adults, these needs may be particularly salient, as age-related functional decline and chronic conditions can erode confidence and perceived control over health behaviors. Care partners play a pivotal role in supporting these motivational needs by providing encouragement, reinforcing progress, and fostering a sense of shared purpose in behavior change [24,25]. Prior studies demonstrate that caregiver involvement can enhance adherence to physical activity, rehabilitation, and self-management behaviors among older adults, particularly when support is autonomy-enhancing rather than directive or coercive [26,27,28]. Despite this, few digital health interventions explicitly incorporate care partners into motivational messaging or examine how motivational content is perceived differently by older adults and their care partners.

The purpose of this study is twofold. First, we aimed to validate motivational text messages designed using the SDT framework to promote physical activity among older adults and their care partners. Second, we sought to evaluate differences in the perceived motivational value of these text messages between older adults and care partners. By including both older adults and their care partners, this study addresses a key gap in digital health interventions, which typically focus only on older adults and rarely consider how motivational messages are interpreted differently across these groups. Findings from this validation study will inform the refinement of motivational messaging strategies to enhance engagement in physical activity among older adults and their care partners.

## 2. Materials and Methods

### 2.1. Study Design and Population

This study is one of the foundational validation studies for the Activity Tracking, Care Partner Co-Participation, Text Reminders, Instructional Education, Video-Guided Physical Rehabilitation, and Exercise (ACTIVE) intervention. The ACTIVE pilot study is a two-arm randomized clinical trial designed to improve physical activity among older adults [29]. The study is registered on ClinicalTrials.gov (NCT07321587, Registration Date 23 December 2025) and approved by the Institutional Review Board (IRB #: i25-01158) [30].

For this cross-sectional study, we recruited three groups of participants: (1) instrument experts, (2) adults aged ≥65 years, and (3) adults aged 18 years and older who self-identified as care partners to an older adult. Participants were identified through ResearchMatch, a national, NIH-funded volunteer registry that connects researchers with individuals interested in participating in health-related studies [31]. ResearchMatch includes more than 120,000 volunteers, including over 13,000 adults aged 65 years and older [31]. A recruitment message was disseminated via the ResearchMatch email listserv, and interested individuals provided their contact information. Those who expressed interest received a link to the study survey. Participants first completed an eligibility screening survey and, if eligible, provided informed consent before accessing the full study questionnaire. All responses to the eligibility survey, consent, and survey were collected via Research Electronic Data Capture (REDCap) [32].

### 2.2. Eligibility Criteria

Individuals were deemed qualified to serve as instrument experts if they met all of the following criteria: (1) at least three years of research expertise in public health, health services, aging, physical activity, rehabilitation, or caregiving research; (2) ability to provide feedback in English; and (3) 18 years or older. Experts were excluded if they were unable to evaluate the clarity and relevance of each text message.

Older adults were eligible to participate if they were community-dwelling, aged 65 years or older, able to read and understand English, had internet access, and were willing to complete the survey. Older adult participants were excluded if they self-reported cognitive impairment that would preclude informed participation, had significant uncorrected visual impairment that would prevent comprehension of survey items, or were currently enrolled in another structured physical activity research study.

Care partners were eligible if they were 18 years or older, identified as an informal caregiver (e.g., family member or friend) who provides any amount of regular unpaid weekly support to an older adult aged ≥65 years, could read and understand English, had internet access, and were willing to complete the survey instruments. We excluded care partners who were paid or professional caregivers (e.g., home health aides or nurses), who reported cognitive or communication limitations that would interfere with completing the survey, or who participated in another dyadic research study involving physical activity interventions.

### 2.3. Message Development

We developed motivational text messages through a structured, theory-informed, multi-step process. First, we identified nine activity scenarios that could occur during daily step monitoring among older adults. These scenarios reflected typical patterns observed in wearable activity data and included: (1) exceeding the daily activity goal, (2) meeting the daily target, (3) slightly below target, (4) low activity, (5) no activity data received, (6) activity improvement from the previous day, (7) activity decrease from the previous day, (8) three or more consecutive days of high activity, and (9) three or more consecutive days of low activity.

Next, we used Self-Determination Theory (SDT) as the guiding behavioral framework for message design [33]. SDT is built on the principle that sustained motivation arises when three core psychological needs are supported: (1) autonomy—a sense of choice and control, (2) competence—feeling capable of making progress, and (3) relatedness—feeling supported and connected [34,35]. Similar to prior studies that used SDT in designing text messages [36,37,38], we applied SDT principles to each scenario to ensure that messages were non-judgmental, supportive, and reinforcing of self-efficacy.

Specifically, we ensured that each motivational text message included one or more of the three core tenets of SDT. Autonomy-supportive language offered choice and avoided pressure (e.g., “Try to take a short walk today or move around the house when you can”, “A little movement today can help boost your mood”, and “Would you like to set a small goal for today?”). Competence was reinforced through statements that highlighted progress and ability (e.g., “Great job yesterday! You were extra active”, “Well done meeting your activity goal”, and “You were so close… just a few more steps next time.”). Relatedness was conveyed through warm, supportive language that emphasized partnership (e.g., “Your body and mind thank you”, “Remember to wear your watch today so we can cheer you on”, and “Your commitment is inspiring.”).

Using this approach, we created nine motivational text messages (Table 1), each tailored to its corresponding scenario. Messages were written to acknowledge day-to-day variability in activity, encourage consistent movement, normalize low-activity days, and reinforce positive behavioral patterns. The final messages were concise (≤160 characters), positively framed, and suitable for automated delivery based on daily activity data.

### 2.4. Sample Size Determination

Our recruitment strategy was aimed at supporting several validation studies of the ACTIVE intervention, including motivational text messages, other instrument design and validation, and activity tracking behavior. As such, our sample sizes were intentionally larger than the minimum required for the current text message validation to provide sufficient representation across all validation activities. For the instrument experts, we targeted 10–20 instrument experts consistent with published recommendations for content analysis [39,40].

To ensure our study was adequately powered to detect meaningful differences in perceived motivational scores between older adults and care partners, we calculated the minimum sample size for independent groups using the standard formula for comparing two means (see Equation (1)) [41].

(1) n=2 (Za/2 +Zβ)2σ2 ∆2 

where = $Z_{a/2\;}$ = 1.96, representing the Z-value for 95% confidence; $Z_{\beta }$ = 0.84, representing the Z-value to detect 80% power; σ = 1, representing the standard deviation of scores (calculated as the range divided by 4); and ∆ = 0.5, representing the minimum detectable difference between groups (effect size) [42]. Hence, the minimum sample size for each group is 63. We added 15% to account for non-normality and potential use of medians [43], yielding 73 participants per group. Our actual sample sizes (older adults = 310, care partners = 305) far exceeded these minimum requirements, ensuring robust content validation and adequately powered comparisons of perceived motivational scores.

### 2.5. Data Analysis

We extracted sociodemographic and health characteristics of all study participants, including age, sex, race/ethnicity, educational attainment, marital status, and self-rated health, as potential confounders, consistent with prior literature. We report frequency distributions and summary statistics among instrument experts, older adults, and care partners.

### 2.6. Content Analysis

We performed a content analysis of each motivational text message by computing the item content validity indices (I-CVI) and Cohen’s Kappa [39,44]. We used the I-CVI to assess the relevance and clarity of each motivational text message for the situation in which it was used. Each of the instrument experts assessed the relevance of the items in the scale on a four-point ordinal scale (1-irrelevant, 2-unable to assess relevance without revision, 3-relevant but needs minor alteration, 4-relevant). Also, the instrument experts assessed the clarity of each motivational text message on a four-point ordinal scale (1-not clear, 2-somewhat clear but needs major revision, 3-mostly clear but needs minor alteration, 4-extremely clear). We recoded the relevance and clarity scales into binary variables—relevant (scores 3 and 4) or not relevant (scores 1 and 2), and clear (scores 3 and 4) or not clear (scores 1 and 2). I-CVI is the proportion of relevant agreement on each item, and it was computed as the number of relevant or clear responses divided by the number of experts [39]. Cohen’s kappa provided inter-rater agreement. With p_o_ representing the observed proportion of agreement, Cohen’s kappa was determined using the formula (p_o_ − 0.5)/(1 − 0.5) [44]. We retained an item if the I-CVI was 0.7 or higher (high validity index) and Cohen’s kappa was 0.6 or higher (good to excellent expert agreement) [39].

### 2.7. Perceived Motivation

Perceived motivational value for each text message was assessed using a 5-point Likert scale ranging from 1 (not at all motivating) to 5 (extremely motivating), with higher scores indicating stronger perceived motivational value. Enrolled older adults and care partners completed the survey and provided ratings for each message. For every text message, we calculated the median and interquartile range (IQR) of the perceived motivational value scores. To compare perceived motivational value scores between older adults and care partners, we used the Mann–Whitney U test, given the non-parametric distribution of the scores, with significance set at *p* < 0.05. Also, we conducted quantile regression to evaluate whether differences in motivational scores persisted after adjusting for sociodemographic and health characteristics, including age, sex, race/ethnicity, educational attainment, marital status, and self-rated health. All analyses were conducted in STATA version 16 [45].

### 2.8. Human Subjects Research

This study was reviewed and approved by the NYU Langone Health Institutional Review Board (IRB#: i25-00450, 21 August 2025). All participants received and signed electronic informed consent before accessing the survey instruments. All study procedures complied with ethical standards for human subject research and the principles outlined in the Declaration of Helsinki.

## 3. Results

### 3.1. Participant Characteristics

Fourteen content experts participated in the study (Mean [SD] age = 30.4 [5.2] years). Eight experts (57%) were male, three (21%) were non-Hispanic White (21%), 10 (71%) were non-Hispanic Black, and one (7%) was Hispanic. The experts represented diverse professional backgrounds, including eight physicians (54%), two nurses (14%), three health service researchers (23%), and two public health researchers (14%). Their research experience ranged from four to 11 years.

A total of 310 older adults, with a mean (standard deviation (SD)) age of 70.1 (4.3) years, were enrolled in the study (Table 2). The older adults were predominantly female (57%), non-Hispanic White (51%), married (69%), and had excellent self-rated health status (68%). Similarly, we enrolled 305 care partners with a mean (SD) age of 35.3 (10.1) years. The care partners were predominantly female (53%), non-Hispanic White (35%), married (78%), and had excellent self-rated health status (77%).

### 3.2. Expert Validation

Regarding relevance, all nine motivational messages demonstrated excellent content validity (Table 3). Item-level CVIs ranged from 0.86 to 1.00, with corresponding kappa values from 0.72 to 1.00, supporting retention of all messages. Regarding clarity, the nine motivational messages had excellent content validity, with item-level CVIs ranging from 0.93 to 1.00 (kappa values from 0.86 to 1.00).

### 3.3. Perceived Motivation

Among older adults, all nine messages had a median rating of 4, indicating they are very motivating (Table 4). Similarly, among care partners, all nine messages had a median rating of 4. Bivariate analysis comparing older adult and care partner scores showed no significant differences in the median scores except for three messages: M5 (*p* = 0.005)—“Looks like we missed your activity data yesterday. No worries—remember to wear your watch today so we can cheer you on!”, M7 (*p* = 0.026)—“Yesterday was a little slower than the day before—and that’s okay. A little movement today can help boost your mood and health”, and M9 (*p* = 0.010)—“We noticed it’s been quiet few days. Would you like to set a small goal for today? A 5-min stretch or short stroll counts!” However, there were no significant differences in perceptions between older adults and care partners in the univariate model or after adjusting for age, sex, race/ethnicity, educational attainment, marital status, and self-rated health.

## 4. Discussion

This study evaluated and validated nine motivational text messages designed to encourage physical activity among older adults and their care partners. Expert assessment supported the content validity of all messages, with high item-level content validity indices indicating that the messages were relevant, clear, and suitable for promoting behavioral motivation. Among end users, both older adults and care partners rated the text messages as very motivational, underscoring their potential to support engagement in physical activity through brief, low-cost digital communication. Together, these findings demonstrate that the messages are acceptable, contextually appropriate, and ready for pilot testing in future behavioral intervention studies.

Although the text messages were rated as highly motivational, the median score of 4 (“very motivating”) suggests that further refinements could enhance their effectiveness. Some participants may prefer more personalized or situationally adaptive messages—for example, incorporating individualized activity goals, progress feedback, or culturally tailored language. For example, instead of a generic message like “Great job yesterday! You were extra active—your body and mind thank you. Keep that energy going today!”, a personalized version might say, “Great job yesterday, Maria—you exceeded your 5000-step goal even on a rainy day. Your body and mind thank you. Keep that energy going today!” While such tailoring can make messages feel more relevant, supportive, and connected to the person’s lived experience [46,47], it must be limited to participants’ wishes and must be accurate; otherwise, it risks being demotivating and cliched [48,49]. Prior studies suggest that personalized motivational messages are more likely to sustain motivation and promote self-efficacy [46,48].

The absence of significant differences between older adults and care partners in perceived motivational value, based on the adjusted regression model, suggests similar perceptions of motivation between the two groups. This alignment may reflect shared goals related to maintaining functional independence. From a behavioral standpoint, care partners often act as both facilitators and co-participants in health-promoting activities [50]. Hence, messages that emphasize encouragement, shared accountability, and mutual reinforcement may appeal to both roles equally. However, it is important to consider alternative explanations for these null findings. The study was powered to detect medium effect sizes, so smaller, potentially meaningful differences could have gone undetected. Additionally, the sample consisted primarily of healthy individuals, which may have limited variability in scores and introduced a ceiling effect. Social desirability or the absence of dyadic interactions may also have attenuated detectable differences.

While the present study focused on initial perceptions of motivational value, the long-term effects of repeated text messaging warrant careful consideration. Although regular prompts can reinforce habits and sustain engagement, message fatigue or habituation may occur over time, diminishing perceived motivational impact [51]. Prior behavioral research suggests that message effectiveness tends to plateau or diminish with increased frequency due to message fatigue [52,53]. On the other hand, for some users, particularly those with low baseline motivation or limited social support, consistent text messaging may serve as a valuable external cue that reinforces accountability and fosters a sense of connectedness. Future longitudinal studies should therefore examine how message frequency, timing, and personalization interact to influence sustained engagement, physical activity adherence, and user satisfaction over extended periods.

This study has several limitations. First, the cross-sectional design captures perceived motivation at a single point in time, precluding conclusions about long-term engagement or behavioral outcomes. Second, participants were primarily healthy older adults; perceptions may differ among individuals with chronic illness, mobility limitations, or cognitive impairment. Third, reliance on self-reported ratings may introduce social desirability and response bias [54,55], particularly given the study’s motivational framing. Lastly, we did not enroll older adult–care partner dyads, and dyadic dynamics may meaningfully shape motivation, decision-making, and perceived support for physical activity in ways that cannot be captured through individual reports. Despite these limitations, the study has notable strengths. The inclusion of instrument experts, older adults, and care partners ensured that messages were evaluated from both theoretical and end-user perspectives. In addition, the motivational text messages were theoretically grounded, enhancing their behavioral relevance and potential to support sustained engagement. These results provide a solid foundation for subsequent pilot testing and for the development of adaptive, technology-enabled physical activity interventions for older adults and their care partners. Future studies will examine racial and ethnic differences in message perception and pilot these messages within a digital intervention to assess its acceptability among older adults with more diverse health conditions, including chronic illness, mobility limitations, and cognitive impairment.

## 5. Conclusions

We present nine motivational text messages with high content validity and perceived motivational value, designed to promote physical activity among older adults and their care partners. While our findings affirm the messages’ acceptability and theoretical soundness, future studies should explore refinements, such as adaptive tailoring, personalization, and variation in delivery, as well as the long-term effects of repeated messaging on sustained motivation and engagement. Overall, these results provide a solid foundation for developing scalable, technology-based interventions that leverage motivational messaging to improve physical activity and health outcomes in older adults and care partners.

## Figures and Tables

**Table 1:** List of motivational text messages.

Item ID	Condition (When to Use)	Message
M1	Excellent day (Exceeded step/activity goal (e.g., 6000+ steps)	“Great job yesterday! You were extra active—your body and mind thank you. Keep that energy going today!”
M2	Met daily target (Achieved target (e.g., 5000 steps)	“Well done meeting your activity goal yesterday! Every step makes a difference for your health. Let’s keep it up!”
M3	Slightly below target (~4000–4999 steps)	“You were so close to your activity goal yesterday—just a few more steps next time. You’ve got this!”
M4	Low activity (Less than 4000 steps)	“We all have slower days sometimes. Try to take a short walk today or move around the house when you can. Every bit counts.”
M5	No activity data (No data received)	“Looks like we missed your activity data yesterday. No worries—remember to wear your watch today so we can cheer you on!”
M6	Activity improved from previous day (Positive change)	“Great news—you moved more yesterday than the day before! Small changes add up. Keep that momentum going!”
M7	Activity decreased from previous day (Negative change)	“Yesterday was a little slower than the day before—and that’s okay. A little movement today can help boost your mood and health.”
M8	Consistently active over 3+ days (active more the 3 days)	“You’ve been on a roll! Three active days in a row—fantastic! Your commitment is inspiring.”
M9	Consistently inactive over 3+ days (inactive more than 3 days)	“We noticed it’s been quiet for a few days. Would you like to set a small goal for today? A 5-min stretch or short stroll counts!”

**Table 2:** Sociodemographic Characteristics of older adults and care partners.

Variables	Older Adults (*n* = 310)	Care Partners (*n* = 305)	*p*-Value
Mean (SD) Age	70.1 (4.3)	35.3 (10.1)	<0.001
Sex			
Male	133 (42.9)	144 (47.2)	0.283
Female	177 (57.1)	161 (52.8)	
Race/Ethnicity			
Non-Hispanic White	157 (50.7)	107 (35.1)	<0.001
Non-Hispanic Black	98 (31.6)	90 (29.5)	
Hispanic	35 (11.3)	92 (30.2)	
Other Races	20 (6.5)	16 (5.3)	
Educational Attainment			
High School or less	257 (82.9)	241 (79.0)	0.039
Some College	47 (15.2)	46 (15.1)	
Bachelor’s or higher	6 (1.9)	18 (5.9)	
Marital Status			
Married	214 (69.0)	239 (78.4)	<0.001
WDS	82 (26.5)	31 (10.2)	
Never Married	14 (4.5)	35 (11.5)	
Self-rated Health			
Excellent	211 (68.1)	234 (76.7)	0.052
Very good/Good	74 (23.9)	55 (18.0)	
Fair/Poor	25 (8.1)	16 (5.3)	

WDS: Widowed, Divorced, Separated.

**Table 3:** Content validity assessment of nine motivational text messages for relevance and clarity among instrument experts (*n* = 14).

Items	E1	E2	E3	E4	E5	E6	E7	E8	E9	E10	E11	E12	E13	E14	No in Agreement	I-CVI	Kappa	Decision
Relevance to Motivation																		
M1	1	0	1	1	1	1	1	1	1	1	1	1	1	1	13	0.93	0.86	Retain
M2	1	1	1	1	1	1	1	1	1	1	1	1	1	1	14	1	1	Retain
M3	1	1	1	1	1	1	1	1	1	1	1	1	1	1	14	1	1	Retain
M4	1	1	1	1	1	1	1	1	1	1	1	1	1	1	14	1	1	Retain
M5	1	1	0	1	1	1	1	0	1	1	1	1	1	1	12	0.86	0.72	Retain
M6	1	1	1	1	1	1	1	1	1	1	1	1	1	1	14	1	1	Retain
M7	1	1	1	1	1	1	1	0	1	1	1	1	1	1	13	0.93	0.86	Retain
M8	1	1	1	1	1	1	1	1	1	1	1	1	1	1	14	1	1	Retain
M9	1	1	0	1	1	1	1	0	1	1	1	1	1	1	12	0.86	0.72	Retain
Clarity of Text Messages																		
M1	1	0	1	1	1	1	1	1	1	1	1	1	1	1	13	0.93	0.86	Retain
M2	1	1	0	1	1	1	1	1	1	1	1	1	1	1	13	0.93	0.86	Retain
M3	1	1	1	1	1	1	1	1	1	1	1	1	1	1	14	1	1	Retain
M4	1	1	1	1	1	1	1	1	1	1	1	1	1	1	14	1	1	Retain
M5	1	1	1	1	1	1	1	0	1	1	1	1	1	1	13	0.93	0.86	Retain
M6	1	1	1	1	1	1	1	1	1	1	1	1	1	1	14	1	1	Retain
M7	1	1	1	1	1	1	1	0	1	1	1	1	1	1	13	0.93	0.86	Retain
M8	1	1	1	1	1	1	1	1	1	1	1	1	1	1	14	1	1	Retain
M9	1	1	1	1	1	1	1	0	1	1	1	1	1	1	13	0.93	0.86	Retain

E: Experts; M: Motivational Texts.

**Table 4:** Difference in the perceived motivational value of each text message among older adults and care partners.

Item ID	All Population (*n* = 615)	Older Adult (*n* = 310)	Care Partner (*n* = 305)	*p*-Value *	Unadjusted Median Difference	Adjusted Median Difference
	Median (IQR)	Median (IQR)	Median (IQR)		(95% CI)	(95% CI)
M1	4.0 (3.0–5.0)	4.0 (3.0–5.0)	4.0 (3.0–5.0)	0.823	0.0 (−0.17, 0.17)	0.0 (−0.24, 0.24)
M2	4.0 (3.0–5.0)	4.0 (3.0–5.0)	4.0 (3.0–5.0)	0.141	0.0 (−0.35, 0.35)	0.0 (−0.62, 0.62)
M3	4.0 (3.0–5.0)	4.0 (3.0–5.0)	4.0 (3.0–5.0)	0.402	0.0 (−0.17, 0.17)	0.0 (−0.49, 0.49)
M4	4.0 (3.0–5.0)	4.0 (3.0–5.0)	4.0 (3.0–5.0)	0.146	0.0 (−0.17, 0.17)	0.0 (−0.40, 0.40)
M5	4.0 (3.0–5.0)	4.0 (3.0–5.0)	4.0 (3.0–5.0)	0.005	0.0 (−0.17, 0.17)	0.0 (−0.73, 0.73)
M6	4.0 (3.0–5.0)	4.0 (3.0–5.0)	4.0 (3.0–5.0)	0.105	0.0 (−0.17, 0.17)	0.0 (−0.28, 0.28)
M7	4.0 (3.0–5.0)	4.0 (3.0–5.0)	4.0 (3.0–5.0)	0.026	0.0 (−0.17, 0.17)	0.0 (−0.45, 0.45)
M8	4.0 (3.0–5.0)	4.0 (3.0–5.0)	4.0 (3.0–5.0)	0.857	0.0 (−0.17, 0.17)	0.0 (−0.24, 0.24)
M9	4.0 (3.0–5.0)	4.0 (3.0–5.0)	4.0 (3.0–5.0)	0.010	0.0 (−0.17, 0.17)	0.0 (−0.51, 0.51)

M: Motivational Text; *p*-value determined from Mann–Whitney U Test; Median difference assessed using quantile regression. Adjusted model: controlled for age, sex, race/ethnicity, marital status, and self-rated health. * *p*-value: statistical significance set at <0.05.

## Data Availability

The original data presented in the study are openly available in FigShare at doi:10.6084/m9.figshare.30801278.

## Abbreviations

The following abbreviations are used in this manuscript:

CI	Confidence Interval
U.S.	United States
WDS	Widowed, Divorced, Separated
